# Supplementary material for: Workplace impact on employees: A Lifelines Corona Research Initiative on the return to work
Source: PLoS One. 2023 Jan 5;18(1):e0279902. doi: 10.1371/journal.pone.0279902 (PMC9815632; doi:10.1371/journal.pone.0279902)
Supplement: Attachment — Submitted filename: Review_01_11_2022.docx [file pone.0279902.s001.docx]

**“Workplace impact on employees: a Lifelines Corona Research Initiative upon a return to work”**

This is a very interesting study, it investigates whether employees working from home had less favourable scores for concern and facilities upon return to work, but better scores for work quality and health than non-home workers. The results show that home workers had less favourable scores for concern and on-site facilities upon return to work, but better scores for work quality and health than non-home workers.

The paper employed a Mann-Whitney U, logistic regression, and Wilcoxon matched-pairs .

**General Comments:**

The paper has some errors regarding tenses, and grammar. Please consider editing the language thoroughly.

**Abstract:**

The abstract lacks a sentence or two setting the background or the rationale for this study. Why should you carry out the study? Why should the reader be interested in reading the paper?

**Introduction:**

- The introduction should be restructured to capture the background, statement of the problem, current literature, existing gaps, the aim of the study and peculiar contributions of this study.
- Please also include the last paragraph in the introduction which explains the structure of the paper. As it is difficult to know what to expect in the paper.

**Methodology:**

- Please provide a thorough justification of the method used. The authors state the methods used, however, they do not provide a justification for the choice of method. Please prove a paragraph of method justification.

Overall, this paper is important and very interesting. However, the paper is not publishable in its current state. The paper needs to be well structured, especially the introduction. A thorough language editing should also be carried out. Subject to making the necessary adjustments I recommend its publication.
